# A Role for Bcl-2 in Notch1-Dependent Transcription in Thymic Lymphoma Cells

**DOI:** 10.1155/2012/435241

**Published:** 2012-01-26

**Authors:** Ronit Vogt Sionov, Shlomit Kfir-Erenfeld, Rachel Spokoini, Eitan Yefenof

**Affiliations:** The Lautenberg Center for General and Tumor Immunology, Institute for Medical Research Israel-Canada (IMRIC), Hebrew University-Hadassah Medical School, Jerusalem 91120, Israel

## Abstract

Notch1 is a transcription factor important for T-cell development. Notch1 is active in double negative (DN) thymocytes, while being depressed in double positive (DP) thymocytes. Synchronously, the expression of Bcl-2 becomes downregulated during the transition from DN to DP thymocytes. We previously observed that overexpression of an intracellular active Notch1 (ICN) in Bcl-2-positive 2B4 T cells leads to the transcription of Notch1-regulated genes. However, these genes were not induced in Bcl-2-negative DP PD1.6 thymic lymphoma cells overexpressing ICN. Here we show that, when Bcl-2 is simultaneously introduced into these cells, Notch-regulated genes are transcribed. Only in the presence of both Bcl-2 and ICN, PD1.6 thymic lymphoma cells become resistant to glucocorticoid (GC)-induced apoptosis. Our data suggest that Bcl-2 plays a role in modulating Notch1 function in T cells.

## 1. Introduction

Notch1 signaling plays a critical role in promoting cell growth, proliferation, and survival of immature T cells [1]. In the thymus, Notch signals are critical throughout the double negative (DN) stages for the maintenance of T-cell specification and for continued differentiation of *αβ* T cells past the *β*-selection checkpoint [2]. Upon interaction with its ligands (e.g., Delta-like 1, Delta-like 4, Jagged 1, and Jagged 2), the Notch1 protein undergoes two proteolytic events, leading to the release of the intracellular Notch domain (ICN). Subsequently, ICN translocates to the nucleus and activates transcription of target genes through its association with C-promoter binding factor 1-recombination binding protein J*κ* (CBF1-RBPJ*κ*) and various members of the Mastermind family [2].

Notch signaling is active in DN, CD4^+^, and CD8^+^ single positive (SP) thymocytes, while being repressed in CD4^+^8^+^ double positive (DP) thymocytes [3, 4]. Similarly, DN, CD4^+^, and CD8^+^ SP thymocytes express elevated levels of Bcl-2, whereas DP thymocytes express low levels of Bcl-2 [5, 6]. Both Notch1 [3, 7, 8] and Bcl-2 [9–11] confer resistance to glucocorticoid (GC)-induced apoptosis. However, this resistance is partial as prolonged exposure to GCs leads to apoptosis of immature T cells overexpressing either Notch1 or Bcl-2 [8–10]. Bcl-2 is an antiapoptotic protein that regulates apoptosis along the intrinsic mitochondrial apoptosis pathway [12]. The alterations in Notch signaling and Bcl-2 expression during thymocyte development may explain the extreme susceptibility of DP thymocytes to GC-induced apoptosis, while DN and SP thymocytes are relatively resistant [13].

The 2B4 T hybridoma and PD1.6 DP thymic lymphoma cell lines are well known to be highly sensitive to GC-induced apoptosis with more than 50% cell death within 20 hrs of incubation with 100 nM dexamethasone (Dex) [3, 7, 8, 10, 14]. The PD1.6 cells were derived from DP thymocytes by immortalization with Radiation Leukemia Virus (RadLV) and have been characterized to represent this stage of thymocyte development [15]. While the mature 2B4 T hybridoma cells express basal Bcl-2 levels [3, 8], the immature DP PD1.6 thymic lymphoma cells barely express any Bcl-2 or Bcl-X_L_[8, 10, 16]. The active intracellular form (ICN) of the transcription factor Notch1 is well documented to confer GC resistance upon lymphoma cells and 2B4 T cells [3, 7]. Interestingly, we observed that overexpression of ICN conferred GC resistance on 2B4 T cells, but not on DP PD1.6 thymic lymphoma cells [8]. Gene expression analysis revealed that the Notch-regulated genes Hes1 and Deltex1 are transcribed in ICN-overexpressing 2B4 cells, but not in ICN-overexpressing PD1.6 cells [8]. Also, overexpression of ICN conferred GC resistance in 2B4, but not in PD1.6 cells [8]. Proteomic studies showed that 2B4 cells express endogenous Bcl-2, with induction of the Bcl-2 member Mcl-1 upon ICN overexpression [8]. The latter may be due to activation of Akt in ICN-overexpressing 2B4 cells [7, 8], a protein kinase that regulates Mcl-1 expression [17]. However, PD1.6 cells, similar to DP thymocytes, do not express any of the three antiapoptotic Bcl-2 members Bcl-2, Bcl-X_L_, or Mcl-1 [8, 10, 16].

In the present study we show that simultaneous expression of Bcl-2 and ICN in PD1.6 cells caused induction of Hes1 and Deltex1 transcription, with concomitant acquisition of GC resistance. These data suggest that Bcl-2 affects Notch1-mediated transcription, and both proteins need to be present for conferring strong GC resistance.

## 2. Material and Methods

### 2.1. Cells

CD4^+^CD8^+^ DP PD1.6 thymic lymphoma cells [18] and CD4^−^8^−^ DN S49 thymic lymphoma cells (kindly provided by A. Hochman, The Hebrew University of Jerusalem, Israel) were grown in DMEM (4.5 g/l glucose) supplemented with 10% heat-inactivated fetal calf serum (FCS), 2 mM glutamine, 10 mM HEPES, 1 mM sodium pyruvate, nonessential amino acids, and 50 *μ*M *β*-mercaptoethanol. PD1.6 overexpressing ICN of Notch1 was prepared as previously described [8]. Bcl-2 overexpression was achieved by electroporating (250 V, 950 *μ*F) the cells with the pSSFV-neo-Bcl-2 plasmid (kindly provided by Dr. Javier Léon, Santander, Spain) followed by selection in 1.5 mg/mL G418 (Sigma) as described [10]. Several clones were analyzed.

### 2.2. Reagents

Dexamethasone (Dex) was purchased from Sigma and dissolved in ethanol at 10^−2 ^M.

### 2.3. Determination of Cell Death

The extent of cell death was determined by propidium iodide (PI) uptake which was analyzed by flow cytometry in combination with altered SSC/FSC scatter as described [8, 10]. We have previously shown that this assay provides better representation for the extent of lymphoma cell death than cell cycle analysis [8, 10]. PI uptake was performed with fresh cell cultures. The cells were harvested, washed in PBS and 5 *μ*g/mL PI added prior to analysis on flow cytometry.

### 2.4. Reverse Transcriptase-Polymerase Chain Reaction (RT-PCR)

Total RNA was isolated from the cells using TRI Reagent (MRC Molecular Research Center) and cDNA prepared by RevertAid first-strand cDNA synthesis kit (Fermentas) using M-MuLV reverse transcriptase and oligo(dT)_18_. PCR was performed using Taq polymerase (Fermentas) and the following primers to: mouse GR forward: GGAAAAGCCATTGTCAAAAGG; and reverse: TGGCCCTCTAGAGACCACAT; mouse Deltex1 forward: GTAAGGCTTCA-AGGGGTCGCT; and reverse: CTCAGCTTGATGCGTGTATAGCC; mouse Hes1 forward: GCCAGTGTCAACACGACACCGG; and reverse: TCACCTCGTTCATG-CACTCG; and mouse glyceraldehyde-3-phosphate dehydrogenase (GAPDH) forward: GGAGCCAAACGGGTCATCATCTC; and reverse: GAGGGG-CCATCCACAGTCTTCT.

### 2.5. Western Blot

Total lysate was prepared by lysing 5 × 10^6^ cells in 250 *μ*L Laemmli protein sample buffer × 1.5. The following antibodies were applied: Bcl-2 (PC68) (diluted 1 : 500) from Calbiochem; phospho-Tyr216 GSK3 (612312) (diluted 1 : 1000) from BD Transduction Laboratories (Franklin Lakes, NJ), phospho-Ser473 Akt (catalog no. 9271) (diluted 1 : 500), phospho-Ser21/9 GSK3*α*/*β* (catalog no. 9331) (diluted 1 : 1000), and phospho-Ser211 GR (catalog no. 4161) (diluted 1 : 1000) from Cell Signaling Technology (Danvers, MA), GR (M20) (diluted 1 : 1000) from Santa Cruz Biotechnology, Inc. (Santa Cruz, CA), Notch1 (mN1A) (diluted 1 : 1000), and *α*-tubulin (DM1A) (diluted 1 : 20,000) from Sigma.

### 2.6. Statistical Analysis

Statistical analysis was performed using Student's *t*-test for paired data. A *P* value less than 0.05 was considered statistically significant. Each experiment was repeated at least 3 times.

## 3. Results

### 3.1. Bcl-2 Supports Notch-1 Transcriptional Function in Thymic Lymphoma Cells

**Figure 1:**
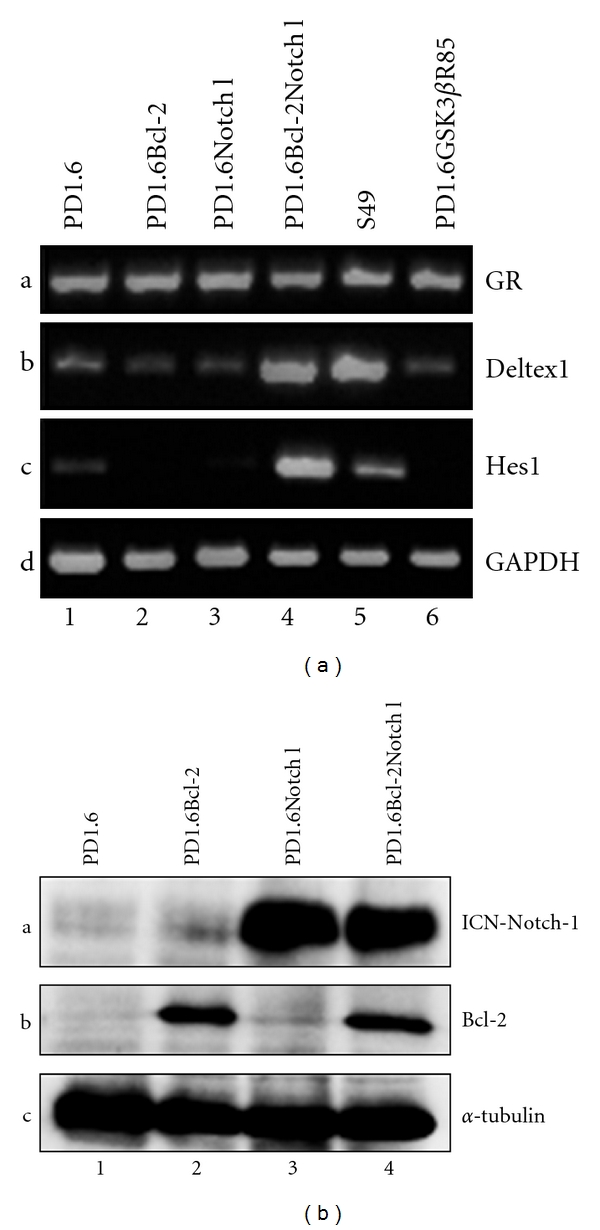
(a) Induction of Deltex1 and Hes1 expression in PD1.6 cells overexpressing both ICN-Notch1 and Bcl-2. Reverse transcriptase-polymerase chain reaction (RT-PCR) analysis of the indicated cells. GAPDH was used as loading control. (b) Western blot analysis of Bcl-2 and Notch1 expression in PD1.6, PD1.6Bcl-2, PD1.6Notch1, and PD1.6Bcl-2Notch1 cells. *α*-Tubulin was used as loading control.

We previously observed that, while overexpressing intracellular active Notch1 (ICN) in 2B4 cells confers resistance to GC-induced apoptosis [8], ICN overexpression in PD1.6 cells (PD1.6Notch1) had barely any effect on GC susceptibility [8]. An ensuing question was why does not ICN confer GC resistance on PD1.6 cells? In contrast to 2B4 cells, where ICN induces gene expression of the Notch1 target genes Deltex1 and Hes1 [8], ICN does not affect expression of these genes in ICN-expressing PD1.6 cells (Figure 1(a) lane 2 and [8]). This indicates that Notch1 signaling is depressed in the DP PD1.6 cells, which fits with the observation that Notch1 signaling is downregulated during the transition from DN to DP thymocytes [3, 4]. The question is why is not Notch1 active in the DP thymic cells? Concomitant with the reduced Notch1 function, the Bcl-2 and Bcl-X_L_ expression levels are downregulated upon transition from DN to DP thymocytes and reactivated again upon transition to SP T cells [5, 6]. This prompted us to test whether overexpression of Bcl-2 could have any effect on Notch1 function. To this end, we overexpressed Bcl-2 in either PD1.6 or PD1.6Notch1 cells (Figure 1(b)). Indeed, we observed that overexpression of Bcl-2 in PD1.6Notch1 cells led to activation of the Notch target genes Deltex1 and Hes1 (Figure 1(a), lane 4), whereas, as expected, Bcl-2 overexpression alone did not activate these genes (Figure 1(a), lane 3). As a positive control for Deltex1 and Hes1 expression, we used the immature DN S49 thymic lymphoma cells (Figure 1(a), lane 5) that endogenously express Bcl-2 and active Notch1 [8]. PD1.6Bcl-2Notch1 cells show similar Deltex1 expression as S49 cells, but a significantly higher level of Hes1 (Figure 1(a), compare lane 4 with lane 5). As a negative control, we included another PD1.6 transfectant that harbors the dominant negative GSK3 plasmid (PD1.6GSK3*β*R85, described in Spokoini et al. [8]). As expected, no activation of the given Notch target genes is observed (Figure 1(a), lane 6). It should be noted that all PD1.6 transfectants contain similar mRNA levels of the glucocorticoid receptor (GR) as untransfected PD1.6 cells (Figure 1(a), Panel a). Taken together, these data show that Bcl-2 can activate Notch1 transcriptional function in DP thymic cells.

### 3.2. PD1.6Bcl2Notch1 Cells Are Resistant to GC-Induced Apoptosis

**Figure 2:**
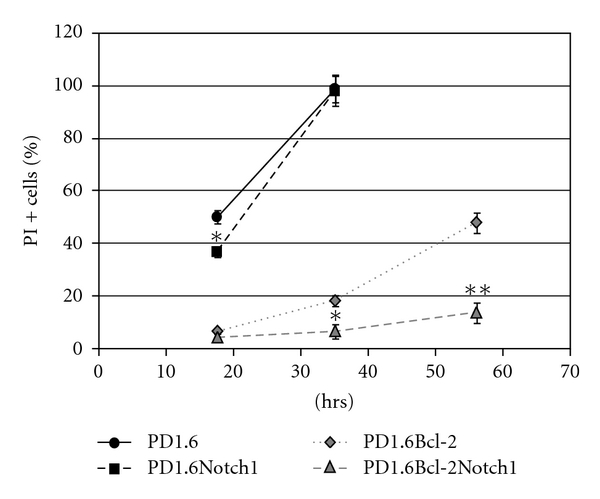
PD1.6 cells overexpressing both ICN-Notch1 and Bcl-2 become resistant to dexamethasone (Dex)-induced apoptosis. The cells were exposed to 100 nM Dex for the indicated time periods, followed by analysis of propidium iodide (PI) positive cells on flow cytometry. Untreated cells were used as control. *P < 0.04  **P < 0.01.

**Figure 3:**
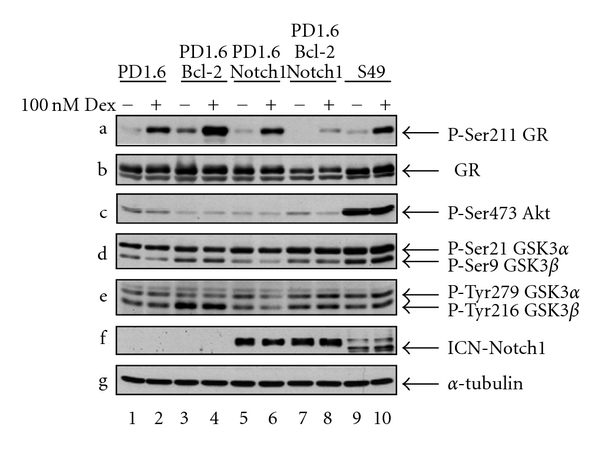
PD1.6Bcl-2Notch1 cells show reduced GR expression. Western blot analysis of untreated or Dex-treated (100 nM, 2 hrs) cells. *α*-Tubulin was used as loading control.

Since ICN overexpression alone had barely an effect on the susceptibility of PD1.6 cells to GC-induced apoptosis ([8] and Figure 2), it was of interest to study the response of PD1.6Bcl2Notch1 to Dex. As can be seen in Figure 2, the expression of both Bcl-2 and Notch1 conferred resistance to 100 nM Dex even after 64 hrs. Bcl-2 overexpression alone led to a delayed apoptotic response, with almost no cell death after 20 hrs, but an increasing apoptotic response after 40 and 64 hrs (Figure 2). These findings support the hypothesis that Bcl-2 and Notch1 cooperate in conferring GC resistance. One of the antiapoptotic functions of Notch has been linked to activation of the PI3K-Akt signaling pathway [19]. While Akt is activated in 2B4Notch1 cells [7, 8], it is not activated in PD1.6Bcl-2Notch1 cells (Figure 3, panel c, lanes 7-8). Thus, the acquisition of GC resistance in these cells is not related to Akt activation. In this context, it should be mentioned that PD1.6 cells do not express PTEN, a phosphatase that inhibits the PI3K-Akt signaling pathway (unpublished data). Nevertheless Akt is not activated by Notch1, suggesting that another mechanism prevents Akt activation in these cells. Moreover, PD1.6 cells overexpressing Bcl-2 show a slight increase in both serine and tyrosine phosphorylation of the Akt target GSK3 *α*/*β* (Figure 3, panels d-e, lanes 3-4 and 7-8). The phosphorylation of Ser21/Ser9 is known to inhibit the activity of GSK3 *α*/*β*. The increase in GSK3 serine phosphorylation may contribute to GC resistance, as GSK3 is essential for GC-induced apoptosis [8]. More profoundly, the resistance seems to be related to reduced GR expression (Figure 3, panel b, lanes 7-8) and a reduced amount of Ser211-phosphorylated GR (Figure 3, panel a) that is important for its nuclear effects [20]. The reduced GR expression may be related to elevated expression of Hes1, a transcription factor known to downregulate GR expression [21]. Also, Deltex-1 has been shown to confer GC resistance in thymocytes [22]. The ICN level is unaffected by Dex treatment (Figure 3, panel f). Combining our data, we propose that Bcl-2 cooperates with Notch1 to confer GC resistance in PD1.6 cells by promoting Notch1-mediated transcription of Hes1 and Deltex1.

## 4. Discussion

In this paper we provide evidence that Bcl-2 affects Notch1 function in immature thymic lymphocytes. In DP thymic cells, Notch1 is unable to induce its target genes Deltex1 and Hes1 in the absence of Bcl-2. When Bcl-2 is coexpressed, Notch1 transcriptional function is activated. This is accompanied by the acquisition of GC resistance. This concords with findings showing that both Deltex1 and Hes1 contribute to GC resistance [21, 22]. Another appreciated antiapoptotic function of Notch is linked to the activation of the PI3K-Akt pathway [19]. While in ICN-overexpressing 2B4 cells Akt is activated [7, 8], there is no indication for Akt activation in PD1.6Bcl-2Notch1 cells. The reason for the inability of Notch1 to activate Akt in these cells is not known, but could be related to high Csk activity (unpublished data). Thus, another mechanism is likely to be responsible for Notch1-induced GC resistance. A protrusive feature of PD1.6Bcl-2Notch1 cells is the reduction in both GR expression and GR Ser211 phosphorylation. The reduced GR expression may be related to the elevated Hes1 expression in these cells. Hes1 has been shown to downregulate GR expression in acute T lymphoblastic leukemia [21]. Since sufficient GR expression level is required for GC-induced apoptosis [13], the reduction in GR expression and Ser211 phosphorylation by Notch-1 overexpression is likely to contribute to GC resistance.

 Interestingly, in the original paper describing a role for Notch1 in conferring GC resistance [3], overexpressing ICN in AKR1010 lymphoma cells led to the induction of Bcl-2. Thus, there may be a mutual cooperation between Notch1 and Bcl-2. A recent study by Wang et al. using pancreatic carcinoma cells has also raised the hypothesis that Bcl-2 can affect Notch1 transcriptional function [23]. These authors showed that TW-37, an inhibitor of Bcl-2, attenuated Notch1-mediated Hes1 transcription. Moreover, overexpression of Bcl-2 increased Hes1 expression, while siRNA to Bcl-2 reduced Hes1 expression in the pancreatic cancer cells studied [23].

 Our observation that Bcl-2 is required for Notch1 function in thymic cells is important as it may explain the alterations in Notch1 function and GC susceptibility during thymocyte development. In DN and SP thymocytes Bcl-2 expression and Notch1 transcriptional activity is high, while it is low in DP thymocytes. Our data have also implications for lymphoblastic leukemia therapy and may explain why repressing either Bcl-2 expression (e.g., by an Bcl-2 inhibitor) or Notch function (e.g., by *γ*-secretase inhibitors) may individually be sufficient for sensitizing the cancerous cells to GC-induced apoptosis [11, 21, 24, 25].

## Abbreviations

Dex: Dexamethasone
DN: Double negative (CD4^−^8^−^)
DP: Double positive (CD4^+^8^+^)
GC: Glucocorticoids
GR: Glucocorticoid receptor
ICN: Intracellular domain of Notch1
SP: Single positive (CD4^+^8^−^ or CD4^−^8^+^).

## References

[1] Aster JC, Pear WS, Blacklow SC (2008). Notch signaling in leukemia. *Annual Review of Pathology*.

[2] Paganin M, Ferrando A (2011). Molecular pathogenesis and targeted therapies for NOTCH1-induced T-cell acute lymphoblastic leukemia. *Blood Reviews*.

[3] Deftos ML, He YW, Ojala EW, Bevan MJ (1998). Correlating notch signaling with thymocyte maturation. *Immunity*.

[4] Hasserjian RP, Aster JC, Davi F, Weinberg DS, Sklar J (1996). Modulated expression of NOTCH1 during thymocyte development. *Blood*.

[5] Moore NC, Anderson G, Williams GT, Owen JJT, Jenkinson EJ (1994). Developmental regulation of bcl-2 expression in the thymus. *Immunology*.

[6] Veis DJ, Sentman CL, Bach EA, Korsmeyer SJ (1993). Expression of the Bcl-2 protein in murine and human thymocytes and in peripheral T lymphocytes. *Journal of Immunology*.

[7] Sade H, Krishna S, Sarin A (2004). The anti-apoptotic effect of Notch-1 requires p56^lck^-dependent, Akt/PKB-mediated signaling in T cells. *Journal of Biological Chemistry*.

[8] Spokoini R, Kfir-Erenfeld S, Yefenof E, Sionov RV (2010). Glycogen synthase kinase-3 plays a central role in mediating glucocorticoid-induced apoptosis. *Molecular Endocrinology*.

[9] Hartmann BL, Geley S, Löffler M (1999). Bcl-2 interferes with the execution phase, but not upstream events, in glucocorticoid-induced leukemia apoptosis. *Oncogene*.

[10] Kfir S, Sionov RV, Zafrir E, Zilberman Y, Yefenof E (2007). Staurosporine sensitizes T lymphoma cells to glucocorticoid-induced apoptosis: role of Nur77 and Bcl-2. *Cell Cycle*.

[11] Ploner C, Rainer J, Niederegger H (2008). The BCL2 rheostat in glucocorticoid-induced apoptosis of acute lymphoblastic leukemia. *Leukemia*.

[12] Frenzel A, Grespi F, Chmelewskij W, Villunger A (2009). Bcl2 family proteins in carcinogenesis and the treatment of cancer. *Apoptosis*.

[13] Sionov RV, Spokoini R, Kfir-Erenfeld S, Cohen O, Yefenof E (2008). Mechanisms regulating the susceptibility of hematopoietic malignancies to glucocorticoid-induced apoptosis. *Advances in Cancer Research*.

[14] Sionov RV, Cohen O, Kfir S, Zilberman Y, Yefenof E (2006). Role of mitochondrial glucocorticoid receptor in glucocorticoid-induced apoptosis. *Journal of Experimental Medicine*.

[15] Zilberman Y, Yefenof E, Katzav S, Dorogin A, Rosenheimer-Goudsmid N, Guy R (1999). Apoptosis of thymic lymphoma clones by thymic epithelial cells: a putative model for ‘death by neglect’. *Immunology Letters*.

[16] Sionov RV, Kfir S, Zafrir E, Cohen O, Zilberman Y, Yefenof E (2006). Glucocorticoid-induced apoptosis revisited: a novel role for glucocorticoid receptor translocation to the mitochondria. *Cell Cycle*.

[17] Wei G, Twomey D, Lamb J (2006). Gene expression-based chemical genomics identifies rapamycin as a modulator of MCL1 and glucocorticoid resistance. *Cancer Cell*.

[18] Zilberman Y, Zafrir E, Ovadia H, Yefenof E, Guy R, Sionov RV (2004). The glucocorticoid receptor mediates the thymic epithelial cell-induced apoptosis of CD4^+^8^+^ thymic lymphoma cells. *Cellular Immunology*.

[19] Gutierrez A, Look AT (2007). NOTCH and PI3K-AKT pathways intertwined. *Cancer Cell*.

[20] Wang Z, Frederick J, Garabedian MJ (2002). Deciphering the phosphorylation "code" of the glucocorticoid receptor *in vivo*. *Journal of Biological Chemistry*.

[21] Real PJ, Tosello V, Palomero T (2009). *γ*-secretase inhibitors reverse glucocorticoid resistance in T cell acute lymphoblastic leukemia. *Nature Medicine*.

[22] Jang J, Choi YI, Choi J (2006). Notch1 confers thymocytes a resistance to GC-induced apoptosis through Deltex1 by blocking the recruitment of p300 to the SRG3 promoter. *Cell Death and Differentiation*.

[23] Wang Z, Azmi AS, Ahmad A (2009). TW-37, a small-molecule inhibitor of Bcl-2, inhibits cell growth and induces apoptosis in pancreatic cancer: involvement of notch-1 signaling pathway. *Cancer Research*.

[24] Heidari N, Hicks MA, Harada H (2010). GX15-070 (obatoclax) overcomes glucocorticoid resistance in acute lymphoblastic leukemia through induction of apoptosis and autophagy. *Cell Death and Disease*.

[25] Li M, Chen F, Clifton N (2010). Combined inhibition of notch signaling and Bcl-2/Bcl-xL results in synergistic antimyeloma effect. *Molecular Cancer Therapeutics*.

